# Snow cover and extreme winter warming events control flower abundance of some, but not all species in high arctic Svalbard

**DOI:** 10.1002/ece3.648

**Published:** 2013-06-29

**Authors:** Philipp R Semenchuk, Bo Elberling, Elisabeth J Cooper

**Affiliations:** 1 Institute for Arctic and Marine Biology, University of TromsøN-9037, Tromsø, Norway; 2 University Center in Svalbard UNISN-9071, Longyearbyen, Norway; 3 Department of Geosciences and Natural Resource Management, Center for Permafrost (CENPERM), University of CopenhagenDK-1350, Copenhagen, Denmark

**Keywords:** Cassiope tetragona, climate change, Dryas octopetala, extreme event, growing season, mild periods, reproductive effort, snow depth, snow fence, Spitsbergen

## Abstract

The High Arctic winter is expected to be altered through ongoing and future climate change. Winter precipitation and snow depth are projected to increase and melt out dates change accordingly. Also, snow cover and depth will play an important role in protecting plant canopy from increasingly more frequent extreme winter warming events. Flower production of many Arctic plants is dependent on melt out timing, since season length determines resource availability for flower preformation. We erected snow fences to increase snow depth and shorten growing season, and counted flowers of six species over 5 years, during which we experienced two extreme winter warming events. Most species were resistant to snow cover increase, but two species reduced flower abundance due to shortened growing seasons. *Cassiope tetragona* responded strongly with fewer flowers in deep snow regimes during years without extreme events, while *Stellaria crassipes* responded partly. Snow pack thickness determined whether winter warming events had an effect on flower abundance of some species. Warming events clearly reduced flower abundance in shallow but not in deep snow regimes of *Cassiope tetragona*, but only marginally for *Dryas octopetala*. However, the affected species were resilient and individuals did not experience any long term effects. In the case of short or cold summers, a subset of species suffered reduced reproductive success, which may affect future plant composition through possible cascading competition effects. Extreme winter warming events were shown to expose the canopy to cold winter air. The following summer most of the overwintering flower buds could not produce flowers. Thus reproductive success is reduced if this occurs in subsequent years. We conclude that snow depth influences flower abundance by altering season length and by protecting or exposing flower buds to cold winter air, but most species studied are resistant to changes.

Winter warming events, often occurring together with rain, can substantially remove snow cover and thereby expose plants to cold winter air. Depending on morphology, different parts of the plant can be directly exposed. On this picture, we see *Dryas octopetala* seed heads from the previous growing season protrude through the remaining ice layer after a warming event in early 2010. The rest of the plant, including meristems and flower primordia, are still somewhat protected by the ice. In the background we can see a patch of *Cassiope tetragona* protruding through the ice; in this case, the whole plant including flower primordia is exposed, which might be one reason why this species experienced a loss of flowers the following season. Photograph by Philipp Semenchuk.

## Introduction

Observed and projected climate change, especially pronounced in Arctic regions, suggest future increase of air temperature and precipitation rates, thereby influencing snow depth, density and duration of snow cover (Serreze et al. 39; ACIA 1; Serreze and Francis 38; IPCC 21; Førland et al. 10). Together with increasing air temperatures, these changes are already provoking responses from some ecosystems, for instance changes in carbon and nutrient cycling, and “shrubification” in Arctic and alpine ecosystems (Sturm et al. 45; Parmesan 31). Climate change also increases the frequency and magnitudes of extreme climatic events (Hansen et al. 13), which can lead to winter warming events and associated reductions in snow cover during winter (Shabbar and Bonsal 40; IPCC 21). These warming events can be asso-ciated with heavy rainfall, as was the case in this study, which can be very effective in removing and compacting snow. Specifically, projected earlier snow melt and increased frequency and magnitude of extreme climatic events, in the form of warming periods and rain events during winters in the high Arctic, could have long-term effects on plant community composition. Frost damage can occur by exposing plants to unexpectedly low air temperatures through removing snow in mid-winter or by exposure to spring frosts due to very early snow melt (Inouye 17; Bokhorst et al. 4, 5; Preece et al. 32). However, increased solid precipitation during years without winter warming events might increase snow depth and thereby delay the onset of the growing season, thus protecting plants, while naturally occurring inter-annual differences in spring temperatures might delay or advance snow melt.

Many arctic-alpine plant species produce flower buds in the year prior to flowering; these then overwinter in a variety of developmental stages (Bliss 3). For that reason growing conditions in the year of bud production, such as growing season length and air temperature, are partly responsible for flower abundance and therefore for a given species' reproductive success (Inouye et al. 20; Inouye and Saavedra 19; Körner 23; Høye et al. 16). However, some species initiate flower primordia during the same year that they flower, and therefore their flower abundance might depend mostly on the current year's growing conditions. Snow cover has been recognized as one of the main drivers for plant growing conditions in the Arctic, and its inter-annual variability is well documented (Hinkler et al. 15). To test the role of snow cover on flowering as a proxy for reproductive success, studies with multi-year monitoring of response variables during differing natural snow conditions are needed. Although several arctic/alpine snow manipulation experiments exist, only a few of these exceed 3 years duration, and even fewer consider inter-annual flower abundance fluctuations (Wipf and Rixen 51). The aim of this study is to fill that gap.

Snow depth controls the duration of snow lie and thereby length of the growing season (Walker et al. 47; Borner et al. 6; Wipf 50; Wipf and Rixen 51; Cooper et al. 7). Early snowmelt and resulting longer growing seasons may be favorable for flower bud production due to potentially higher energy and photosynthate accumulation throughout the summer. However, snow cover also directly controls soil and canopy temperatures during winter and spring, thereby protecting arctic and alpine plants from damaging sub-zero temperatures. Premature snow melt in spring, as well as shallow snow cover or snow melt during winter caused by extreme warming events, expose above ground tissues to detrimental winter and spring frosts. This negatively affects flower buds and substantially reduces flower abundance in subsequent growing seasons through freezing, desiccation, or deacclimation without sufficient reacclimation (Gates 11; Firmage and Cole 9; Larcher 24; Høye et al. 16; Bokhorst et al. 4; Inouye 18). Many processes control snow depth and melt out timing and lead to large spatial and temporal variations in arctic snow cover (Hinkler et al. 15). These processes would therefore affect flower abundances, and species-specific responses would be expected due to specific physiological parameters and growth requirements. For instance, a species with greater frost hardiness would lose fewer flower buds in the case of exposure to extremely low temperatures than a species with low frost hardiness.

This study was originally intended to experimentally assess the role of timing of spring snow melt (and thus the length of growing season) on flower abundance for a set of common high-arctic plant species. Our initial hypothesis was that an experimentally delayed spring snow melt will reduce flower abundance. We also expected that the responses would be species specific. However, during 5 years of monitoring flower abundance in the study site, we experienced two extreme warming events during mid-winter which exceeded normal warm periods in the study area, and we opportunistically report these here with the post-hoc hypothesis that deeper snow would prevent plants from being exposed to winter air. Thus, we present a combination of both a manipulation and observation study which was not originally intended to include winter warming events. For some species we observed that deeper snow cover buffered plants from extreme winter warming events and saved the subsequent flower crop in one case, but not in the other case. Here, we present species specific responses of flower abundance to (1) snow melt timing, and (2) extreme winter warming events under contrasting snow depths.

## Materials and Methods

### Study site

The study site is situated in Adventdalen, about 12 km east of Longyearbyen, western Spitsbergen (78°17′N, 16°07′E), and spans an area of approximately 2 km^2^ in the valley to the south west of Advent river. The vegetation is dominated by the two evergreen dwarf shrubs *Dryas octopetala* and *Cassiope tetragona*, and the deciduous dwarf shrub *Salix polaris*. For more details see Cooper et al. (7). Annual mean air temperature and precipitation during the reference period 1961–1990 at Longyearbyen airport (14 km NW of the study site) is −6.7°C and 190 mm and snow depth ranged from 0 to around 35 cm (Førland et al. 10; Norwegian meteorological institute, www.eklima.met.no). The background snow conditions at the study site were similar to those observed at the airport (Morgner et al. 27).

### Experimental setup

To test the influence of snow depth on flower abundance, 12 snow fences (6 m long and 1.5 m high) were erected in autumn 2006, perpendicular to the prevailing winter wind direction along the valley from south-east. These fences serve as topographical features reducing wind speed on their lee side, thereby depositing suspended snow and creating a snow patch of 1.5 m depth, that is the height of the fences, at the deepest point. Resulting snow patches were approximately 20–30 m long, with snow depth decreasing linearly with distance from the fences. For more details see Cooper et al. (7). Data used for this study was collected 2008–2012.

The following four snow depth regimes were investigated using a combination of natural variation in topography and experimental manipulation, thereby creating a snow depth gradient from very shallow to very deep snow.

1. *Shallow*: unmanipulated snow cover with naturally very shallow snow (approx. 1–5 cm deep), usually on slight ridges which were wind-blown. These tended to melt out first.
2. *Normal*: natural unmanipulated snow cover (10–35 cm deep), representative of most of the study area. These usually became snow-free after *Shallow*.
3. *Medium*: experimentally increased snow cover (approx. 60–100 cm deep); approx. 10–20 m behind fences, melted out after *Normal* and before *Deep*.
4. *Deep*: experimentally increased snow cover (approx. 150 cm deep); *c*. 3–12 m behind fences; this was the last regime to become snow-free.
5. 12 plots (approximately 50 × 50 m) with 2–6 sub-plots (75 × 75 cm) per treatment arranged in four blocks (with three plots each) were used to compare the snow regimes. *Shallow*: two sub-plots per plot; *Normal*: six sub-plots per plot; *Medium*: three subplots per plot; *Deep*: six subplots per plot.

### Observations

Flowers of six species (*Bistorta vivipara*,* Cassiope tetragona*,* Dryas octopetala*,* Pedicularis hirsuta*,* Saxifraga oppositifolia*,* Stellaria crassipes* ssp. *confusa*) were counted in each subplot at weekly intervals during the whole snow-free period. These species were chosen because they are the most common non-graminoid species in the study area, that is their flowers were easily countable in the field. Flower counts started in 2008 in *Normal* and *Deep*, and in 2010 in *Shallow* and *Medium*, and continued in all regimes until the end of flowering in September 2012.

It has been reported that a majority of arctic-alpine species, such as the ones studied here, produce preformed flower buds (Sørensen 44 as cited in Bliss 3). Except for *Saxifraga oppositifolia*, which produces very mature floral buds towards the end of the growing season (Larl and Wagner 25), we are not aware of more detailed studies on flower preformation in the species studied here.

Percentage coverage in each subplot of *C. tetragona* and *D. octopetala* was visually estimated at peak season in 2011, and this data is used to represent the whole study period, assuming stable coverage. Soil surface temperature at around 1 cm below surface in *Normal* and *Deep* was measured hourly by temperature loggers (Gemini Data Loggers, Tinytag, UK) installed in each of the 12 plots, in total 24 loggers, during the whole study period. Temperature loggers in *Medium* and *Shallow* were installed in autumn 2010 and in three of the 12 fences only. Hourly air temperature data from the Adventdalen weather station run by the University Centre of Svalbard (UNIS) about 6.5 km north-west of the study site in the same valley was used (downloaded from www.unis.no). Daily snow depth and precipitation data from Longyearbyen airport was obtained from the Norwegian meteorological institute (www.eklima.met.no).

Melt out dates of individual sub-plots were observed daily from mid-May until the end of snow melt in 2010–2012. The date at which 50% of each subplot was snow free was recorded. In 2008 and 2009 (2 years with observations covering only *Normal* and *Deep*), snow melt date was estimated by visual comparison of soil surface temperature profiles and associated melt out dates from 2010 to 2012. Snow melt in a given sub-plot usually occurred a certain number of days after the associated soil temperature logger measured a plateau at around 0°C, that is the zero curtain (Kelley and Weaver 22). This was consistent during 2010–2012, and the zero curtain observations from 2008 to 2009 were used to estimate snow melt date for these years.

### Statistical analyses

The effects of snow regime on flower abundance were tested statistically for each species separately. Flower abundance was defined as the highest flower count per species, sub-plot and year, that is the flower peak. This definition seemed most useful as opposed to yearly flower sums or means, where flowers might have been counted several times. Since data from only two of the snow regimes (*Normal* and *Deep*) were collected during all 5 years of the experiment (2008–2012), we analyzed the data once with all years (the all-years model) and once with all regimes (the all-regimes model), with the latter using data from 2010 to 2012 only. For the analysis of *Dryas octopetala* and *Cassiope tetragona*, the areal coverage of each species per sub-plot was included as a covariate in the models to account for the influence of species abundance on flower abundance. Areal cover estimation of the other species was low (data not available) and was assumed to be homogenous across sub-plots. An interaction between snow regime and year was tested, since we expected different effects during different years. Species coverage was included as an additive term, since the influence of that covariate can be assumed to be constant across years and snow regimes. The following fixed effects in the full models were used for these analyses: flower abundance ~ snow regime * year + cover.

Flower abundance data were analyzed with linear mixed effects models using R, version 2.15.0 (R Development Core Team 33). Mixed effects were defined as nested sub-plots within plots within blocks as random intercepts (random = ~1block/plot/sub-plot). Counts of flowers as response were modeled as a Poisson distribution using the *glmmPQL* function of the MASS package, which is taking potential over-dispersion into account. Measurements were done on the same sub-plot and same plant individuals each year and natural fluctuations due to changes in plant size, life history, abundance, and delayed costs of reproduction might influence flower abundance (see Obeso 29 and references therein). Therefore, we included a term to control for potential correlations between past and current reproduction, as these could mask results we wanted to evaluate with our design (Hamel et al. 12). The autocorrelation term *Phi* of each minimal model is presented in Table S1. Overall, the CI of *Phi* is below 0 in some cases, demonstrating the presence of reproductive trade-offs, whereas in most cases it includes 0 or was positive, suggesting no apparent trade-off. Nevertheless, we kept *Phi* in all models to control for the impact these autocorrelations might have on the estimates we were interested in. Since this study was not aiming at, and does not have the potential to reliably estimate cost of reproduction of the studied species, we will not discuss this parameter further.

Similar analyses were done on the effects of snow regime on melt out dates. Again, all-years and all-regimes models were fitted, assuming an interaction between year and snow regime. The fixed effects in the full models were melt out date ~ snow regime * year. Melt out dates were analyzed with the same random intercepts as flower counts, but under the assumption of drawing the data from a normal distribution. Such, the *lme* function of the nlme package could be used.

Model simplification of all models was made by reverse step wise reduction of the full model including all interactions, until all higher order terms included at least one statistically significant term on the 5% level (i.e., *P*-value ≤ 0.05) (Zuur et al. 52). Predicted values from the Poisson models presented in graphs were back-transformed (log-link) and estimated with mean values of covariates (i.e., species cover) if appropriate.

## Results

### Temperature and snow characteristics

**Figure 1:**
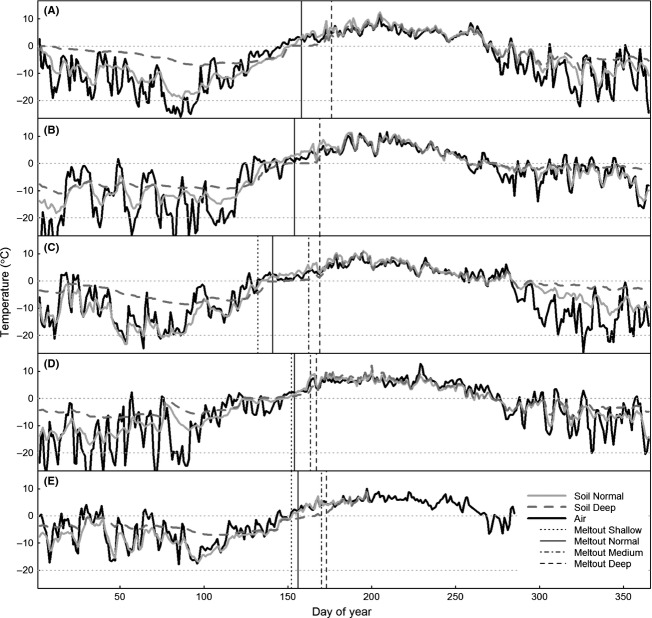
Air (black line) and soil surface temperatures at 1 cm depth in *Normal* (solid grey line) and *Deep* (dashed grey line) snow regimes. Vertical lines show average melt out dates of all observed snow regimes. Air temperatures show daily averages at Longyearbyen airport, Svalbard obtained from the Norwegian Meteorological Institute, and soil surface temperatures show daily averages measured in each snow regime with a total of 24 loggers. *Normal*: unmanipulated snow depth as found representative for most of the study area; *Deep*: manipulated snow depth with snow fences; *Shallow*: unmanipulated snow regime shallower than *Normal* as found on slightly elevated ridges throughout the study area; *Medium*: increased manipulated snow regime shallower than *Deep*. Each panel shows data of 1 year: (A) 2008, (B) 2009, (C) 2010, (D) 2011, (E) 2012.

The mean annual air temperature during the period 2008–2012 was −4.7, 2°C warmer than during the reference period 1961–1990 (Førland et al. 10). During snow free seasons, soil temperatures in all treatments followed air temperature closely. During snowy seasons, soil temperatures were buffered from air temperature, that is soil temperature was more stable than air temperature and did not follow fluctuations closely. This was especially pronounced in *Deep*, where soil temperatures during winter were more stable and usually warmer than both ambient air and soil in *Normal* (see Fig. 1). Snow depth and resulting winter soil temperatures were uniform across plots. Following these observations, we assume that the soil in *Medium* was colder than in *Deep*, but warmer than in *Normal*, and that the soil in *Shallow* was closest to air temperature. This is supported by the additional temperature data from these snow regimes (Fig. S1). Soil temperatures in *Deep* and *Medium* were relatively low (as low as −9°C) compared to snow fence studies in, for instance, Alaska (Schimel et al. 37), probably due to differences in manipulated snow depth and snow quality – snow in our study site is wind packed and compact, thereby offering relatively poor insulation.

Table 1 shows melt out dates of all snow regimes during all years. The average melt out date across all years for *Shallow*,* Normal*,* Medium* and *Deep* was day of year (DOY) 144, 153, 163, and 170. However, the full model including the year * regime interaction was selected for both the all-years and all-regimes model, suggesting significant differences across years. This was mainly due to the fact that in 2010 melt out dates of *Normal* and *Shallow* were not only earlier than the other two snow regimes, but also earlier than during other years.

**Table 1:** Model estimates of melt out dates (day of year DoY) during different years and snow regimes with 95% confidence limits of all years and snow regimes

Snow regime	Year	DoY	Lower	Upper
Normal	2008	158	156	161
Deep	2008	175	172	177
Normal	2009	153	150	156
Deep	2009	170	167	172
Shallow	2010	132	129	135
Normal	2010	142	140	144
Medium	2010	160	158	163
Deep	2010	167	165	169
Shallow	2011	150	147	152
Normal	2011	155	153	157
Medium	2011	162	159	164
Deep	2011	166	164	168
Shallow	2012	151	148	153
Normal	2012	157	155	160
Medium	2012	168	165	170
Deep	2012	171	169	174
Shallow	Mean	144		
Normal	Mean	153		
Medium	Mean	163		
Deep	Mean	170		

The estimates of the all-years and all-regimes models were so similar that the results of both combined are shown here (see text for details). *Normal*: unmanipulated snow depth as found representative for most of the study area; *Deep*: manipulated snow depth with snow fences; *Shallow*: unmanipulated snow regime shallower than *Normal* as found on slightly elevated ridges throughout the study area; *Medium*: increased manipulated snow regime shallower than *Deep*.

**Figure 2:**
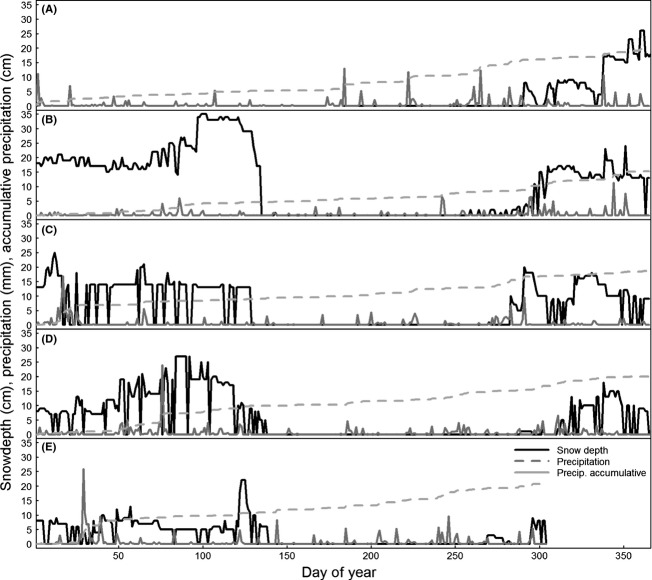
Daily snow depth (solid black line, cm) and precipitation (solid grey line, mm), and accumulative precipitation (dashed grey line, cm) at Longyearbyen airport, Svalbard obtained from the Norwegian Meteorological Institute. Each panel shows data of 1 year: (A) 2008, (B) 2009, (C) 2010, (D) 2011, (E) 2012.

Two pronounced mid-winter warming events were observed during the study period. The first event with positive temperatures was during DOY 14–19 and DOY 21–25 in 2010, the second event occurred during DOY 26–32 and DOY 35–40 in 2012. Both events coincided with abnormally high rainfall, and snow cover disappeared at the meteorological station (Fig. 2) and presumably at most of *Normal* and *Shallow* (data not available). Soil temperature in both *Normal* and *Deep* were close to zero during the warm events. After both warm periods, soil temperatures in *Normal* followed air temperature closely, that is the buffering effect of a snow layer was lost as during snow-free summer periods, while soil temperature in *Deep* remained quite stable until spring thaw. In 2010, soil temperature in *Normal* dropped below −20°C repeatedly, which is the record low recorded during the study period. The remaining part of winter 2012 was abnormally mild, and soil temperatures in *Normal* together with air temperatures did not drop below −16°C, which was a common and reoccurring temperature during the study period.

### Flower abundance

**Figure 3:**
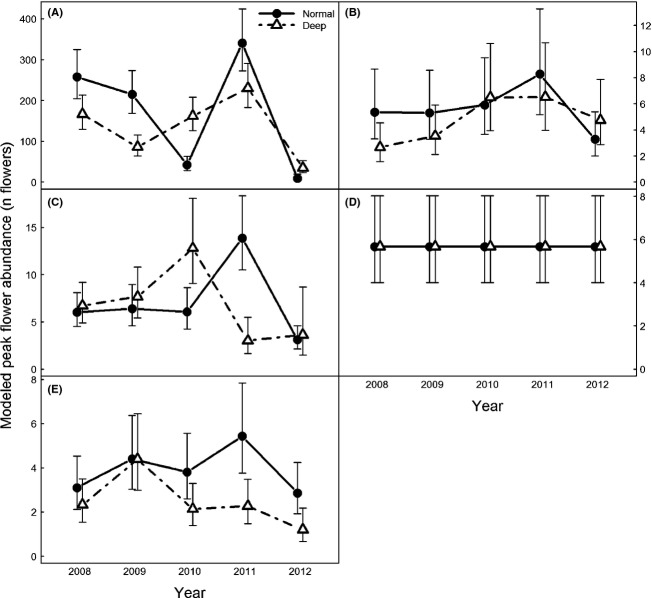
Model estimates of peak flower abundance during different years and in different snow regimes for each species of the “all-years” model, a generalized mixed effects model assuming a Poisson distribution of the response variable (see text for details). Presented are back-transformed estimates (log-link of the model). Error bars show 95% confidence intervals. Note the different scales on the y axes. *Normal*: unmanipulated snow depth as found representative for most of the study area; *Deep*: manipulated snow depth with snow fences. Each panel shows results of one species: (A) *Cassiope tetragona*, (B) *Dryas octopetala*, (C) *Pedicularis hirsuta*, (D) *Saxifraga oppositifolia*, (E) *Stellaria crassipes*.

**Figure 4:**
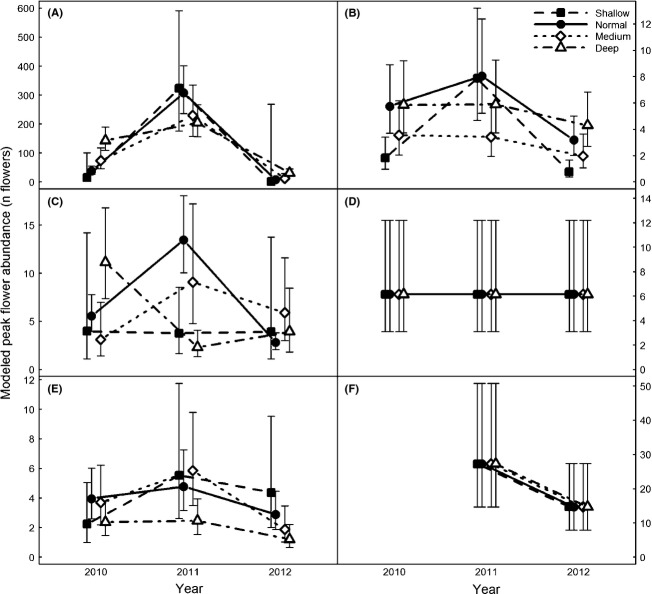
Model estimates of peak flower abundance during different years and in different snow regimes for each species of the “all-regimes” model, a generalized mixed effects model assuming a Poisson distribution of the response variable (see text for details). Presented are back-transformed estimates (log-link of the model). Error bars show 95% confidence intervals. Note the different scales on the y axes. *Normal* and *Deep* as in Fig. 3; *Shallow*: unmanipulated snow regime shallower than *Normal* as found on slightly elevated ridges throughout the study area; *Medium*: increased manipulated snow regime shallower than *Deep*. Each panel shows results of one species: (A) *Cassiope tetragona*, (B) *Dryas octopetala*, (C) *Pedicularis hirsuta*, (D) *Saxifraga oppositifolia*, (E) *Stellaria crassipes*, (F) *Bistorta vivipara*.

In most of the cases, the full model was chosen, that is significant interactions between year and snow regime were found (see Supporting Information Table S1 for model summaries of fixed effects of the selected minimal models). Additionally, all species except *Saxifraga oppositifolia* showed a peak in flower abundance in *Control* in 2011, followed by a trough in 2012. However, back-transformed model estimates shown in Figs. 3 and 4 illustrate that for most species and years the flower abundance differences were not significant or in some cases merely reflect a statistically non-significant trend. We assumed that “statistically significantly different” means that a 95% CI around one mean estimate does not cross the mean value of another estimate and vice versa (Smith 42). Normally, interactions were retained in the minimal model after model selection with AIC if one of the interaction terms was statistically significant. However, biological significance and conclusiveness has to be evaluated individually, which is why we will sometimes refer to statistically non-significant trends in the text. When interpreting the model estimates, it has to be taken into account that the generalized linear mixed effects models used are quite new, and calculations not as exact as other methods (Zuur et al. 52). Therefore, the classical p-value test might not always be in accordance with the estimated 95% confidence intervals.

#### Cassiope tetragona

For *C. tetragona*, both considered models (all-years and all- regimes) resulted in statistically significant interactions between years and regimes (Table S1), that is the estimated mean flower abundance, corrected for plant coverage, was different across years and regimes, ranging from around 1 to 355 per sub-plot. The all-years model estimated that in 2008, 2009, and 2011, abundance in *Deep* was lower than in *Normal*, and that in 2010 and 2012 that relationship was reversed while the abundance in both snow regimes in 2012 was lower than during all other years (see Fig. 3A). The all-regimes model estimated increasing abundances with increasing snow depth in 2010, a reverse trend in 2011, and no difference in 2012. Again, all abundances were comparatively low in 2012, especially in *Deep* and *Medium*, and abundance was highest in 2011, a year without preceding winter warming event (see Fig. 4A).

#### Dryas octopetala

For *D. octopetala*, both considered models (all-years and all-regimes) resulted in statistically significant interactions between years and snow regimes (Table S1), that is the estimated mean flower abundance, corrected for plant coverage, was different across years and regimes, ranging from around 1 to 8 per sub-plot. A trend in the all-years model can be noted, showing initial significant lower abundances in *Deep* than in *Normal* during the first year (2008), which evened out during the remaining 4 years. For *Normal*, 2011 had the highest and 2012 the lowest flower abundance in the whole study period (see Fig. 3B). These peaks and troughs are also somewhat represented in both unmanipulated snow regimes *Shallow* and *Normal* in the all-treatments model, but not in the manipulated regimes *Medium* and *Deep* (see Fig. 4B).

#### Pedicularis hirsuta

For *P. hirsuta*, both considered models (all-years and all-regimes) resulted in statistically significant interactions between years and regimes (Table S1), that is the estimated mean flower abundance was different across years and regimes, ranging from around 2 to 15. This is mainly attributable to a high peak in *Deep* in 2010 and a high peak in *Normal* in 2011 followed by lows the years after, while the abundances during all other years and regimes were relatively constant in both models (see Figs. 3C and 4C).

#### Saxifraga oppositifolia

For *S. oppositifolia*, the Null model was selected for the all-years and the all-regimes model, that is the estimated mean flower abundance per sub-plot did not differ across years and regimes and was estimated as six in both models (see Figs. 3D and 4D; Table S1).

#### Stellaria crassifolia

For *S. crassifolia*, both considered models (all-years and all-regimes) resulted in statistically significant interactions between years and regimes (Table S1), that is the estimated mean flower abundance was different across years and regimes, ranging from around 1 to 6 per sub-plot. Although not statistically significant for 2010 and not significant for all regimes for 2012 in the all-regimes model, both models suggest that abundances in *Deep* were lower than in all other regimes from 2010 but not earlier, whereas the other regimes had similar abundances each year except in 2012, when flower abundances were higher with lower snow depth (see Figs. 3E and 4E). No notable peaks were recognized.

#### Bistorta vivipara

Flower abundance data for *B. vivipara* were only collected for 2011 and 2012, therefore only the all-treatment model was considered for this species and the interaction and regime terms were removed by model selection (Table S1), that is the estimated mean flower abundance was across years, but not regimes, ranging from around 12 to 29. Abundances were higher during 2011 than during 2012 (see Fig. 4F).

## Discussion

Our hypothesis that delayed spring snow melt would reduce flower abundances held for only two of the six observed species. *Stellaria crassipes* had fewer flowers in *Deep* than in all other snow regimes, although that signal is visible only from the 4th year of the experiment. We assume, however, that this species does not produce overwintering flower buds because it (1) produces flowers at the end of new shoots instead of overwintering axillas (own observations), (2) flowers very late in the season (Cooper et al. 7), and (3) is not affected by winter warming events, which might be a trait of some, though not all, chamaephytes with overwintering flower buds, as we will argue for later on. The lower flower abundance of *S. crassipes* in *Deep* can therefore be attributed to insufficient resource allocation to flower production due to a shortened period between onset of growth and flowering, thereby indirectly supporting our hypothesis. That flower abundance was reduced only 4 years after snow manipulation started could be due to possible delayed costs of reproduction or direct fecundity costs, that is growing seasons in *Deep* were not long enough to replenish energy reserves used for previous years' reproduction in the long run (Obeso 29). The fact that this response was not provoked in *Medium* points out that *Deep* crossed a certain threshold for snow melt date.

Of the remaining observed species, only *Cassiope tetragona* showed the hypothesized response to increased snow depth; the later the individuals melted out, the fewer flowers they had in the following season. In 2007, the first summer following snow manipulation, indications for no difference between *Deep* and *Normal* were found (data could not be included in the analysis here due to incomplete observations). The effect of *Deep* increased each year until 2009, that is the snow manipulation effect on *C. tetragona* became more pronounced over the initial years of the study, pointing out an accumulative effect of the previous years' growing conditions; overwintering flower buds of one season might contribute to the pool of flower buds of more than only one following seasons, as shown for *Bistorta vivipara* by Diggle (8). Growing seasons shortened by later snow melt contributed fewer *C. tetragona* flower buds, which might be explained by shorter annual growth increments caused by a shortened (and therefore, in terms of growing degree days colder) growing season, as found by Mallik et al. (26) and Rumpf et al. (in prep) in the same study site, and by Weijers et al. (49) in Ny-Ålesund and Endalen, Svalbard and sub-arctic Sweden. In other studies (Rozema et al. 36; Weijers et al. 48, 49), the number of flower buds formed per year seems to be related to annual shoot length growth, and thus to accumulative summer temperatures (Stef Weijers, pers. Comm.); longer and warmer seasons yielded longer shoots with more leaf axillae, the location where actual flower bud formation occurs. This is confirmed by Mallik et al. (26), who found fewer leaves in *Deep* than in *Normal* after the 2007 growing season, the first year of the study with a shortened growing season, and no difference before treatment allocation. However, during our study, *C. tetragona's* flower abundance response to the snow regimes was overlain by its response to the winter warming events in 2010 and 2012, which will be discussed next.

Winter warming events are common on Svalbard, but are usually not as severe as those observed in early 2010 and in 2012. Accumulated temperature sums and precipitation during January to March throughout 37 years (1976–2012) recorded at Longyearbyen airport show that 2010 and 2012 were among the warmest (fourth warmest and warmest, respectively) and by far the wettest. Temperature sums were 1.4 and 3.1 SD, and precipitation during warming 2.2 and 2.7 SD above the 1976–2012 mean for 2010 and 2012, respectively (data from Norwegian Meteorological Institute, not shown). Following the reasoning of Smith (43), both warming events reported here could be considered as “climate extremes”, while following a more climatological definition the observed warming periods in 2010 and 2012 might be called “warm” and “extremely warm”, respectively, and both events were “very moist”, not “extremely moist” (nomenclature used in Hansen et al. 13). However, not enough data was available to compare our observations with an earlier standard reference period (Norwegian Meteorological Institute), and the fact that our data are based on only one measurement station makes comparison difficult.

Snow-poor or mild winters have been shown to freeze, desiccate, or deharden overwintering meristems and flower buds of berry yielding, ericaceous dwarf shrubs (Raatikainen and Vänninen 34; Ogren 30; Taulavuori et al. 46; Bokhorst et al. 4) and other species (Gates 11; Firmage and Cole 9; Høye et al. 16; Inouye 18; Mallik et al. 26) in sub-arctic and temperate regions and alpine habitats, thereby significantly reducing shoot growth, berry and capsule yield, and flower abundances. Similar effects have been observed on flower abundances in this study for two of the four observed chamaephytes, that is species which keep their overwintering meristems above ground; snow melting by warm temperatures together with rainfall might expose overwintering tissues, which are normally protected by the snowpack, to subsequent cold winter air temperatures and winds which may destroy exposed tissue. Of all our studied species, *C. tetragona* showed the strongest response to winter warming events by significantly reduced flower abundances. In 2010, *C. tetragona* flower abundances in all snow regimes except *Deep*, and in all regimes in 2012 were clearly affected. *Dryas octopetala* responded to these warming events only in the un-manipulated snow regime *Shallow* and *Normal* in 2012, although its response was not as strong as that of *C. tetragona*. The lower the initial snow depth, the higher the proportion of removed snow by warm air temperatures and heavy rain, that is a deep snowpack will last longer than a shallow snowpack. Thus, the severity of flower abundance reduction might have increased with decreasing snow cover in both cases because plants under a deeper snow pack might have still been protected from exposure to detrimental winter temperatures after the warming event by a remaining, sufficiently deep snowpack.

The influence of the observed warming events was stronger on *C. tetragona* than on *D. octopetala*, and the reason for this might be twofold; (1) the shoots of *Cassiope tetragona* are more erect and taller than the procumbent *D. octopetala*. In addition, *C. tetragona* produces its flower buds on the shoot tips. Therefore, *C. tetragona* flower buds might be exposed to colder air temperatures over a longer time period than *D. octopetala*, which keeps its flower buds close to the ground and might be still protected by a remaining layer of snow and ice after mid-winter snow melt by warm events (personal observation). Additionally, the rosette like structure of *D. octopetala* shoot tips might serve as protection for flower buds (Inouye 17). Raatikainen and Vänninen (34) came to similar conclusions on the difference of proportions of surviving flower buds after a particularly snow-poor and cold winter in Finland: *Vaccinium myrtillus* has a high canopy and therefore lower proportion of flower bud survival and *V. vitis-idea* has low canopy and therefore higher proportion of flower bud survival. For the same reason one of the remaining two chamaephytes of this study, that is *Saxifraga oppositifolia* might not have been affected by the warming events; it is of very low stature. Secondly, (b) *Dryas octopetala* is adapted to grow in areas with shallow snow, as opposed to *C. tetragona* which requires a consistent snow cover during winter (Rønning 35). Therefore the smaller effect of warm periods on *D. octopetala* might be not only of morphological, but also of a physiological nature, that is *D. octopetala* might develop stronger frost hardening and withstand cold temperatures better than *C. tetragona*, as found for snow bed species in alpine New-Zealand by Bannister et al. (2).

In 2012, *C. tetragona* individuals in *Deep* were affected by the warming event, unlike in 2010. The 2012 warming event was more severe than the one in 2010, with higher temperatures and greater precipitation, and two possible scenarios might have been responsible for the flower abundance crash in *Deep* during that year. (1) Warm temperatures and rain might have been sufficient to remove enough snow in *Deep* to expose plants to following cold winter air, thereby freezing flower buds to death. This might be possible given the fact that 2012 was a particularly snow-poor year (Fig. 2). However, the winter of 2012 was also relatively warm, and soil temperatures after the warming event never reached abnormally low temperatures, as was the case for *Normal* during long periods in 2010. Therefore, an alternative explanation is possible where (2) the warm temperatures themselves were long and warm enough to deharden overwintering flower buds, thus rendering them susceptible to the subsequent intermediately cold temperatures. Similar mechanisms might have been responsible for the lower flower abundance in all treatments for the hemicryptophyte *Bistorta vivipara* in 2012, although we unfortunately cannot compare with the 2010 event since data is not available for that year.

Unfortunately, we cannot disentangle whether the effect of the warming periods was due to temperature sums, accumulated precipitation, or if both had to be high to cross the threshold of inducing a loss of flower buds. In any case, in order to be considered an “extreme climatic event”, the observed response should be extreme enough to impact the ecosystem severely enough to result in temporary or even permanent community structure changes or similar (Smith 43). This was not the case in our study, where only one species' threshold was clearly exceeded by the warming periods, and its recovery was fast enough to cover the events' effect only one season after. The flower abundance of *C. tetragona* can therefore be described as very resilient, while the other species' flower abundances are resistant to the climate extremes observed here. However, although this study focuses on flowers, it may be reasonable to assume that other above-ground organs may respond in a similar way to shorter growing seasons or exposure to freezing air temperatures through mid-winter mild events (Inouye 17). For instance, survival of overwintering vegetative stages of a monocarpic species was drastically reduced by exposure to cold winter temperatures if thermal insulation was not sufficient enough (Simons et al. 41). Thus, this study may also give a justification for the synchrony of high Arctic herbivore dynamics in relation to wide scale icing events recently reported by Hansen et al. (14).

The hemicryptophyte and semi-parasite *Pedicularis hirsuta* is most likely not affected by the warming events due to below ground overwintering and subsequent protection from cold air temperature. However, it had a flower peak in *Deep* in 2010 and in *Normal* in 2011, the year after particularly early snow melt caused by a winter warm event in the same snow regime. This elongation of the growing season might have facilitated production of either overwintering rhizomes or viable seeds, leading to larger or more individuals the following year yielding more flowers. Similar, although not as pronounced or statistically significant flower peaks were observed for *C. tetragona*,* D. octopetala*, and *S. crassipes*. These peaks were followed by significant crashes of flower abundances, which might indicate direct fecundity costs caused by excessive flowering events the year before (Obeso 29), while a combination of this and winter warming events might have been the case for *C. tetragona* and *D. octopetala*.

Although not examined in this study, the observed effects of season length and winter warming events could have specific effects on the only known annual species on Svalbard overwintering as seeds (*Koenigia groenlandica*). Winter warming might only have an effect if it breaks seed dormancy and thereby reduces the seed bank. Short, late starting seasons could potentially restrict seed set by delaying seed ripening processes too late into autumn, while seasons starting too early could expose seedlings to late spring frosts and thereby not only kill reproductive organs but the whole plant. Both scenarios are also valid for the perennials examined in this study (Inouye 17), however would have stronger implications on annuals, since for those whole individuals and not only vegetative parts of individuals are at risk.

This study fails to estimate what would happen in the case of earlier snowmelt caused by warmer air temperatures during spring, as suggested for the future by climate change models. Repeated trials of snow removal in this study failed because of insufficient marking of sub-plots (marking poles removed by reindeer), or because of wind refilling the removed snow, thereby reducing the number of replicates to a useless level. In any case, artificial snow removal would fall into a period of the year with very low air temperatures and expose protected plants to the cold, thereby confounding the experimental treatment of snow removal with exposure to early season frost. A combination of snow removal and warming is suggested to mimic a natural, earlier snow melt (see also Wipf and Rixen 51).

Given the evidence presented in our study, we conclude: Season length as dictated by snow melt timing has various plant species-specific effects, independent of life-form. Species with overwintering above-ground flower buds (chamaephytes) are affected by winter warm events in various degrees, depending on the positioning of buds, and on the snow depth during winter. An increase of frequency and amplitude of extreme winter warm events will decrease flower abundance and thus reproductive success of some species (here: *Cassiope tetragona*) and thereby favor the fitness of others. This underlines the importance of winter conditions and their influence on summer processes. The impact of potential snow cover changes on high-Arctic plant community composition dynamics caused by altered reproductive success is complex and cannot be answered with the current knowledge of the system; more multi-year, multi-season, and multi-species studies incorporating a set of predictor variables are required to fill this gap.

## Supporting Information

**Table S1.** Raw model outputs of the flower abundance models as presented by the R software. Fixed effects show which covariates and interactions of the full model flowers ~ regime * year (+cover where appropriate) remain in the minimal model. *Phi* is an autocorrelation term included due to potential reproductive trade-offs from previous seasons. Model selection was done by step-wise removal of non-significant terms, until all higher order terms were significant. Only minimal model outputs are shown, that is after model selection. See main text for details about the all-years and all-regimes models.
