# Supplementary material for: Snow cover and extreme winter warming events control flower abundance of some, but not all species in high arctic Svalbard
Source: Ecol Evol. 2013 Jun 29;3(8):2586–99. doi: 10.1002/ece3.648 (PMC3930050; doi:10.1002/ece3.648)
Supplement: Supplementary file 1 — Table S1. Raw model outputs of the flower abundance models as presented by the R software. Fixed effects show which covariates and interactions of the full model flowers ~ regime * year (+cover where appropriate) remain in the minimal model. Phi is an autocorrelation term included due to potential reproductive trade-offs from previous seasons. Model selection was done by step-wise removal of non-significant terms, until all higher order terms were significant. Only minimal model outputs are shown, that is after model selection. See main text for details about the all-years and all-regimes models. [file ece30003-2586-SD1.docx]

**Supporting information**

Table S1

| **Species, model, fixed effects, Phi** | **model terms** | **Value** | **Std.Error** | **DF** | **t-value** | **p-value** |
| --- | --- | --- | --- | --- | --- | --- |
| **Cassiope** | **(Intercept)** | 4,8875 | 0,1573317 | 340 | 31,064951 | 0 |
| **all-years** | **treatincr** | -0,439768 | 0,1493285 | 74 | -2,944971 | 0,0043 |
| **treat * year + cover** | **year9** | -0,183231 | 0,1111474 | 340 | -1,648538 | 0,1002 |
| **CI -0.0151 [-0.1499, 0.1203]** | **year10** | -1,81282 | 0,1985082 | 340 | -9,132215 | 0 |
|  | **year11** | 0,277247 | 0,0985898 | 340 | 2,812128 | 0,0052 |
|  | **year12** | -3,367609 | 0,407347 | 340 | -8,267175 | 0 |
|  | **cover** | 0,015529 | 0,0022149 | 74 | 7,011083 | 0 |
|  | **treatincr:year9** | -0,476609 | 0,1890487 | 340 | -2,521092 | 0,0122 |
|  | **treatincr:year10** | 1,786404 | 0,2352141 | 340 | 7,594801 | 0 |
|  | **treatincr:year11** | 0,049678 | 0,152455 | 340 | 0,325855 | 0,7447 |
|  | **treatincr:year12** | 1,811218 | 0,4593937 | 340 | 3,942627 | 0,0001 |
| **Dryas** | **(Intercept)** | 0,9607009 | 0,26771251 | 447 | 3,588554 | 0,0004 |
| **all-years** | **treatincr** | -0,6987247 | 0,20518556 | 100 | -3,405331 | 0,001 |
| **treat * year + cover** | **year9** | -0,010173 | 0,09740429 | 447 | -0,104441 | 0,9169 |
| **CI 0.2375 [0.1133, 0.3543]** | **year10** | 0,0982503 | 0,10552388 | 447 | 0,931072 | 0,3523 |
|  | **year11** | 0,4347225 | 0,10031762 | 447 | 4,333461 | 0 |
|  | **year12** | -0,4932917 | 0,12800232 | 447 | -3,853771 | 0,0001 |
|  | **cover** | 0,0331949 | 0,00426232 | 100 | 7,787995 | 0 |
|  | **treatincr:year9** | 0,2910754 | 0,17797084 | 447 | 1,635523 | 0,1026 |
|  | **treatincr:year10** | 0,7906415 | 0,18237923 | 447 | 4,335151 | 0 |
|  | **treatincr:year11** | 0,4586038 | 0,18185867 | 447 | 2,52176 | 0,012 |
|  | **treatincr:year12** | 1,0720285 | 0,20518024 | 447 | 5,224814 | 0 |
| **Pedicularis** | **(Intercept)** | 1,7971207 | 0,1521224 | 186 | 11,813649 | 0 |
| **all-years** | **treatincr** | 0,1058192 | 0,2052825 | 105 | 0,515481 | 0,6073 |
| **treat * year** | **year9** | 0,0594207 | 0,1876518 | 186 | 0,316654 | 0,7519 |
| **CI -0.1043 [-0.2713, 0.0688]** | **year10** | 0,0036546 | 0,1899634 | 186 | 0,019239 | 0,9847 |
|  | **year11** | 0,8328095 | 0,1361417 | 186 | 6,117227 | 0 |
|  | **year12** | -0,6610169 | 0,1926843 | 186 | -3,43057 | 0,0007 |
|  | **treatincr:year9** | 0,073588 | 0,2639834 | 186 | 0,27876 | 0,7807 |
|  | **treatincr:year10** | 0,6423837 | 0,260344 | 186 | 2,467442 | 0,0145 |
|  | **treatincr:year11** | -1,6378415 | 0,3421356 | 186 | -4,787113 | 0 |
|  | **treatincr:year12** | 0,0451347 | 0,4974428 | 186 | 0,090734 | 0,9278 |
| **Saxifraga** | **(Intercept)** | 1,735235 | 0,1737344 | 54 | 9,987859 | 0 |
| **all-years** |  |  |  |  |  |  |
| **1** |  |  |  |  |  |  |
| **not available** |  |  |  |  |  |  |
| **Stellaria** | **(Intercept)** | 1,1314913 | 0,1959826 | 249 | 5,773427 | 0 |
| **all-years** | **treatincr** | -0,2892308 | 0,2093617 | 98 | -1,381489 | 0,1703 |
| **treat * year** | **year9** | 0,3492365 | 0,1021759 | 249 | 3,417994 | 0,0007 |
| **CI 0.1332 [-0.07, 0.3258]** | **year10** | 0,2046838 | 0,1145072 | 249 | 1,787519 | 0,0751 |
|  | **year11** | 0,559434 | 0,1012654 | 249 | 5,524432 | 0 |
|  | **year12** | -0,0832921 | 0,1267974 | 249 | -0,656891 | 0,5119 |
|  | **treatincr:year9** | 0,2865001 | 0,1643145 | 249 | 1,743608 | 0,0825 |
|  | **treatincr:year10** | -0,2884822 | 0,2010186 | 249 | -1,435102 | 0,1525 |
|  | **treatincr:year11** | -0,5838777 | 0,1940566 | 249 | -3,008801 | 0,0029 |
|  | **treatincr:year12** | -0,5808535 | 0,2968924 | 249 | -1,956445 | 0,0515 |
| **Cassiope** | **(Intercept)** | 3,0685965 | 0,2179439 | 209 | 14,079751 | 0 |
| **all-regimes** | **treatincr** | 1,3268171 | 0,1966032 | 94 | 6,748704 | 0 |
| **treat * year + cover** | **treatmedium** | 0,6597758 | 0,2764671 | 94 | 2,386453 | 0,019 |
| **CI -0.5354 [-0.6692, -0.3679]** | **treatshallow** | -0,9778619 | 1,0215146 | 94 | -0,957267 | 0,3409 |
|  | **year11** | 2,0900666 | 0,1737671 | 209 | 12,027976 | 0 |
|  | **year12** | -1,5547897 | 0,298748 | 209 | -5,204353 | 0 |
|  | **cover** | 0,0138938 | 0,0023718 | 94 | 5,858009 | 0 |
|  | **treatincr:year11** | -1,736726 | 0,2065851 | 209 | -8,40683 | 0 |
|  | **treatmedium:year11** | -0,9519271 | 0,2950312 | 209 | -3,226531 | 0,0015 |
|  | **treatshallow:year11** | 1,0267263 | 1,0849855 | 209 | 0,946304 | 0,3451 |
|  | **treatincr:year12** | 0,0248136 | 0,3317147 | 209 | 0,074804 | 0,9404 |
|  | **treatmedium:year12** | -0,2669506 | 0,5070984 | 209 | -0,526428 | 0,5991 |
|  | **treatshallow:year12** | -1,1085434 | 2,8514129 | 209 | -0,38877 | 0,6978 |
| **Dryas** | **(Intercept)** | 1,0882687 | 0,24469 | 318 | 4,44754 | 0 |
| **all-regimes** | **treatincr** | 0,0180193 | 0,1781018 | 149 | 0,101174 | 0,9195 |
| **treat * year + cover** | **treatmedium** | -0,4833455 | 0,2430526 | 149 | -1,988646 | 0,0486 |
| **CI 0.161 [-0.0464, 0.3551]** | **treatshallow** | -1,159144 | 0,2889648 | 149 | -4,011367 | 0,0001 |
|  | **year11** | 0,3364722 | 0,0795718 | 318 | 4,228536 | 0 |
|  | **year12** | -0,5919053 | 0,1100617 | 318 | -5,37794 | 0 |
|  | **cover** | 0,031733 | 0,0039735 | 149 | 7,986147 | 0 |
|  | **treatincr:year11** | -0,3320376 | 0,123427 | 318 | -2,690154 | 0,0075 |
|  | **treatmedium:year11** | -0,3756929 | 0,2138001 | 318 | -1,757216 | 0,0798 |
|  | **treatshallow:year11** | 1,1366041 | 0,2326465 | 318 | 4,885541 | 0 |
|  | **treatincr:year12** | 0,2817504 | 0,1560765 | 318 | 1,805207 | 0,072 |
|  | **treatmedium:year12** | 0,0019056 | 0,2765212 | 318 | 0,006891 | 0,9945 |
|  | **treatshallow:year12** | -0,2663529 | 0,3912395 | 318 | -0,680792 | 0,4965 |
| **Pedicularis** | **(Intercept)** | 1,7131841 | 0,1783009 | 95 | 9,608385 | 0 |
| **all-regimes** | **treatincr** | 0,6956513 | 0,2600592 | 95 | 2,674973 | 0,0088 |
| **treat * year** | **treatmedium** | -0,5783995 | 0,4459807 | 95 | -1,296916 | 0,1978 |
| **CI -0.5014 [-0.7095, -0.2128]** | **treatshallow** | -0,3349243 | 0,684999 | 95 | -0,488941 | 0,626 |
|  | **year11** | 0,8858997 | 0,1655549 | 63 | 5,351093 | 0 |
|  | **year12** | -0,6839788 | 0,169208 | 63 | -4,042237 | 0,0001 |
|  | **treatincr:year11** | -2,4545137 | 0,3206957 | 63 | -7,653716 | 0 |
|  | **treatmedium:year11** | 0,1842405 | 0,5233052 | 63 | 0,352071 | 0,726 |
|  | **treatshallow:year11** | -0,9412387 | 0,7969387 | 63 | -1,181068 | 0,242 |
|  | **treatincr:year12** | -0,3562804 | 0,460754 | 63 | -0,773255 | 0,4423 |
|  | **treatmedium:year12** | 1,3248177 | 0,4876361 | 63 | 2,716816 | 0,0085 |
|  | **treatshallow:year12** | 0,664222 | 0,8328193 | 63 | 0,797558 | 0,4281 |
| **Saxifraga** | **(Intercept)** | 1,816027 | 0,3415709 | 40 | 5,316691 | 0 |
| **all-regimes** |  |  |  |  |  |  |
| **1** |  |  |  |  |  |  |
| **CI 0.2921 [-0.2172, 0.6763]** |  |  |  |  |  |  |
| **Stellaria** | **(Intercept)** | 1,3741268 | 0,2193549 | 136 | 6,264399 | 0 |
| **all-regimes** | **treatincr** | -0,5170476 | 0,2364635 | 101 | -2,186585 | 0,0311 |
| **treat * year** | **treatmedium** | -0,0689784 | 0,2605214 | 101 | -0,264771 | 0,7917 |
| **CI -0.2004 [-0.465, 0.0971]** | **treatshallow** | -0,5683199 | 0,4197312 | 101 | -1,354009 | 0,1788 |
|  | **year11** | 0,1885779 | 0,1007506 | 136 | 1,871729 | 0,0634 |
|  | **year12** | -0,3169795 | 0,1045468 | 136 | -3,031939 | 0,0029 |
|  | **treatincr:year11** | -0,1499756 | 0,1893293 | 136 | -0,792141 | 0,4297 |
|  | **treatmedium:year11** | 0,2720761 | 0,1781381 | 136 | 1,527333 | 0,129 |
|  | **treatshallow:year11** | 0,7156812 | 0,3180044 | 136 | 2,250538 | 0,026 |
|  | **treatincr:year12** | -0,3544838 | 0,2558416 | 136 | -1,38556 | 0,1681 |
|  | **treatmedium:year12** | -0,3593273 | 0,2552824 | 136 | -1,407568 | 0,1615 |
|  | **treatshallow:year12** | 0,9864303 | 0,3089274 | 136 | 3,193082 | 0,0017 |
| **Bistorta** | **(Intercept)** | 3,304771 | 0,3173905 | 156 | 10,41232 | 0 |
| **all-regimes** | **year12** | -0,6206 | 0,0608386 | 125 | -10,20076 | 0 |
| **year** |  |  |  |  |  |  |
| **not available** |  |  |  |  |  |  |
