# Supplementary figures and images for: Snow cover and extreme winter warming events control flower abundance of some, but not all species in high arctic Svalbard
Source: Ecol Evol. 2013 Jun 29;3(8):2586–99. doi: 10.1002/ece3.648 (PMC3930050; doi:10.1002/ece3.648)

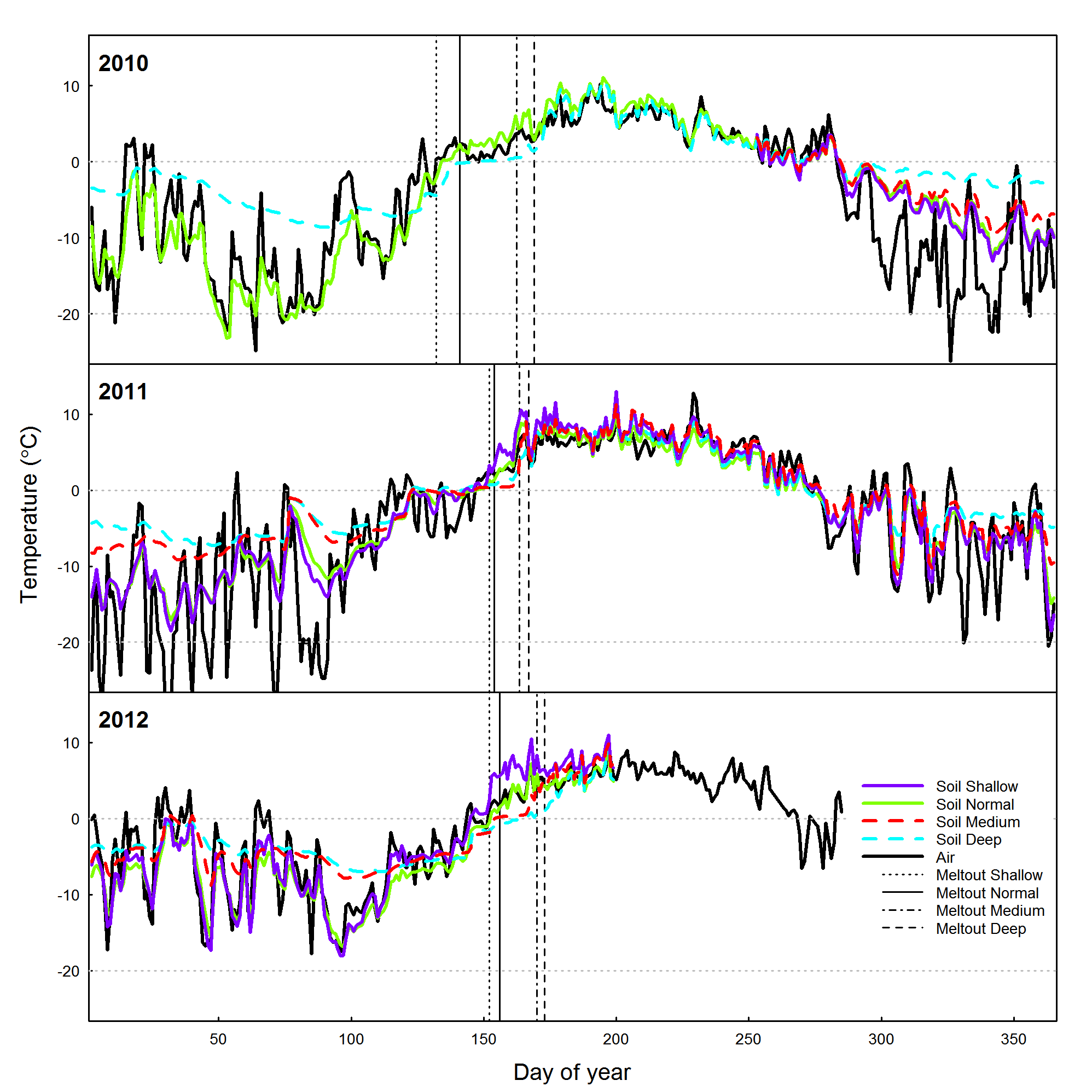

Supplement: Supplementary file 2 [file ece30003-2586-SD2.tiff]
